# Antitumor activity of sorafenib-incorporated nanoparticles of dextran/poly(dl-lactide-*co*-glycolide) block copolymer

**DOI:** 10.1186/1556-276X-7-91

**Published:** 2012-01-27

**Authors:** Do Hyung Kim, Min-Dae Kim, Cheol-Woong Choi, Chung-Wook Chung, Seung Hee Ha, Cy Hyun Kim, Yong-Ho Shim, Young-Il Jeong, Dae Hwan Kang

**Affiliations:** 1 National Research & Development Center for Hepatobiliary Cancer, Research Institute for Convergence of Biomedical Science and Technology, Pusan National University Yangsan Hospital, Beomeo-ri, Mulgeum-eup, Yangsan, 626-770, Republic of Korea; 2 School of Medicine, Pusan National University, Yangsan, 626-770, Republic of Korea; 3 Department of Internal Medicine, Medical Research Institute, Pusan National University School of Medicine and Medical Research Institute, Yangsan, 626-770, Republic of Korea

**Keywords:** sorafenib, polymeric micelle, dextran, poly(DL-lactide-*co*-glycolide)

## Abstract

Sorafenib-incoporated nanoparticles were prepared using a block copolymer that is composed of dextran and poly(DL-lactide-*co*-glycolide) [Dex*b*LG] for antitumor drug delivery. Sorafenib-incorporated nanoparticles were prepared by a nanoprecipitation-dialysis method. Sorafenib-incorporated Dex*b*LG nanoparticles were uniformly distributed in an aqueous solution regardless of the content of sorafenib. Transmission electron microscopy of the sorafenib-incorporated Dex*b*LG nanoparticles revealed a spherical shape with a diameter < 300 nm. Sorafenib-incorporated Dex*b*LG nanoparticles at a polymer/drug weight ratio of 40:5 showed a relatively uniform size and morphology. Higher initial drug feeding was associated with increased drug content in nanoparticles and in nanoparticle size. A drug release study revealed a decreased drug release rate with increasing drug content. In an *in vitro *anti-proliferation assay using human cholangiocarcinoma cells, sorafenib-incorporated Dex*b*LG nanoparticles showed a similar antitumor activity as sorafenib. Sorafenib-incorporated Dex*b*LG nanoparticles are promising candidates as vehicles for antitumor drug targeting.

## Introduction

Nanoparticles have been extensively investigated as a means of specifically targeting drugs to a desirable site of action [1]. Notably, nanoparticles having a hydrophobic inner core and hydrophilic outer shell have received great attention due to their superior properties in drug delivery [2-6]. They are regarded to be ideal vehicles for antitumor drug delivery because their hydrophobic inner core is an appropriate reservoir for hydrophobic anticancer drugs and because their hydrophilic outer shell facilitates avoidance of the reticuloendothelial system, long blood circulation, and the improvement of enhanced permeation and retention [EPR] effect in tumor tissue [6].

Cholangiocarcinoma [CC], a malignant tumor arising from the biliary tract, has a high mortality rate. Even though surgical resection is regarded as a curative method, most of patients diagnosed with a latent CC state are not considered for surgical resection [7]. Furthermore, conventional radiation or chemotherapeutic treatment is known to have limited advantages [7]. Therefore, novel treatment option is required to enhance therapeutic efficacy of CC.

Sorafenib inhibits tumor cell proliferation and vascularization by the activation of the receptor for tyrosine kinase signaling in the Ras/Raf/Mek/Erk cascade pathway [8]. Sorafenib is an effective chemotherapeutic agent against various tumor types including CC [9] and inhibits proliferation, angiogenesis, and invasion of tumor cells [9,10]. However, poor aqueous solubility and undesirable side effects limit the clinical application and local treatment of sorafenib. These side effects might be overcome by use of nanoparticles for tumor delivery and controlled release of sorafenib [11,12].

In this study, we prepared sorafenib-incorporated Dex*b*LG nanoparticles as an antitumor drug delivery system. The properties of sorafenib-incorporated Dex*b*LG nanoparticles were studied in terms of core-shell structure, particle size, morphology, and drug release rate. Antitumor activity of sorafenib-incorporated Dex*b*LG nanoparticles was tested using human cholangiocarcinoma [HuCC-T1] cells.

## Experimental details

### Materials

Dextran from *Leuconostoc *spp. (average molecular weight [MW] approximately 6,000), hexamethylene diamine [HMDA], *N,N*-dicylohexylcarbodiimide [DCC], and *N*-hydroxysuccimide [NHS] were purchased from Sigma-Aldrich (St. Louis, MO, USA). Sorafenib was purchased from LC Laboratories (Woburn, MA, USA). Spectra/Por™ dialysis membranes (MW cutoff [MWCO] = 2,000 g/mol and 8,000 g/mol) were purchased from Spectrum Labs (Rancho Dominguez, CA, USA). Poly(DL-lactic acid-*co*-glycolic acid) (PLGA-5005, MW = 5,000 g/mol) were purchased from Wako Pure Chemicals (Osaka, Japan).

### Synthesis of Dex*b*LG copolymer

Dex*b*LG copolymer was synthesized as reported previously [13]. Aminated dextran was prepared as follows. Dextran (180 mg) dissolved in dimethylsulfoxide [DMSO] was mixed with sodium cyanoborohydride and stirred for 24 h. After that, 10 equivalents of HMDA were added and stirred for 24 h at room temperature. The resulting aminated dextran was obtained by dialysis against deionized water and was lyophilized. *N*-hydroxysuccimide PLGA [PLGA-NHS] was prepared by reaction with DCC and NHS. Dex*b*LG copolymer was prepared by dissolving 120 mg of aminated dextran and 100 mg of PLGA-NHS in DMSO and undergoing reaction for 2 days. Reactants were dialyzed to remove unreacted dextran (MWCO of dialysis membrane = 8,000 g/mol), and the product was lyophilized. The resulting white powder was dissolved in chloroform to remove unreacted PLGA. Yield of the final product was about 89% (*w*/*w*).

### Preparation of sorafenib-incorporated Dex*b*LG nanoparticles

The sorafenib-incorporated Dex*b*LG nanoparticles were prepared by the nanoprecipitation-dialysis method as follows. Dex*b*LG copolymer dissolved in 3 ml of DMSO was mixed with sorafenib in 2 ml of DMSO. This solution was added dropwise to 15 ml of deionized water for over 10 min to form nanoparticles. The solvent was removed by dialysis against deionized water for 1 day. Empty nanoparticles of Dex*b*LG copolymer were prepared by the same procedure, omitting sorafenib. To evaluate the drug contents and loading efficiency, 5 mg of sorafenib-incorporated nanoparticles were distributed into the mobile phase (acetonitrile/methanol/1% acetic acid in a ratio of 35:38:27) and stirred overnight. Drug concentration was determined with high-performance liquid chromatography [HPLC]. The drug content (in percent) was calculated using the following equations:

$$\text{Drug content = }\frac{\text{Drug weight in the nanoparticles}}{\text{Weight of the nanoparticles}}\times 100$$

and

$$\text{Loading eficiency = }\frac{\text{Residual drug in the nanoparticles}}{\text{Initial feeding amount of drugs}}\times 100.$$

### Analysis of nanoparticles

The characterization of nanoparticles was performed in DMSO-d_6 _or D_2_O using 500 MHz^1^H nuclear magnetic resonance [NMR] spectroscopy (500 MHz superconducting FT-NMR spectrometer; Varian Unity-Inova 500; Agilent Technologies, Foster City, CA, USA). The morphology of nanoparticles was observed by transmission electron microscopy [TEM] using a JEM-2000 FX II microscope (JEOL, Tokyo, Japan). One drop of nanoparticle solution containing phosphotungstic acid (0.05% *w*/*w*) was placed onto a carbon film coated on a copper grid for TEM. Observation was done at an accelerating voltage of 80 kV. The particle size and zeta potential were measured by the Nano-ZS apparatus (Malvern Instruments, Malvern, UK). A sample solution prepared by dialysis was used to determine the particle size.

### Drug release study *in vitro*

The release experiment was carried out *in vitro*. A sample solution prepared by dialysis was used directly. This solution was introduced into the dialysis membrane. Next, the dialysis membrane was placed in a 200-ml bottle with 100 ml of phosphate buffered saline [PBS] containing 1% (*v*/*v*) Tween 80 [PBST]. This bottle was placed in a shaking incubator with a stirring speed of 100 rpm at a temperature of 37°C. At specific times, the PBST was sampled for analysis of drug concentration. After each sampling, the entire volume of PBST was replaced with fresh PBST to prevent drug saturation. The concentration of the released sorafenib was determined by HPLC.

### HPLC analysis

The Flexar HPLC system (PerkinElmer, Waltham, MA, USA) was equipped with a Solvent Manager 5-CH degasser, an autosampler, a quaternary LC pump, a column oven, and a UV-visible detector. Chromatography was performed on a guard column (SecurityGuard^® ^Guard Cartridge Kit; Phenomenex, Torrance, CA, USA) and on a C18 column (Brownlee C18^®^, 5 μm, 150 × 4.6; PerkinElmer) at 37°C. Sorafenib was eluted isocratically with mobile phase (acetonitrile/methanol/1% acetic acid at a ratio of 35:38:27) at a flow rate of 1 ml/min and monitored at 254 nm. The chromatograms were recorded and integrated with the Chromera 2.1 system software (PerkinElmer).

### Cell cytotoxicity test *in vitro*

HuCC-T1 cells maintained in RPMI 1640 (10% fetal bovine serum, 5% CO_2 _at 37°C) were used to evaluate the antitumor activity of sorafenib-incorporated nanoparticles. Viability of tumor cells was evaluated by a 3-(4,5-dimethylthiazol-2-yl)-2,5-diphenyltetrazolium bromide [MTT]-based cell proliferation assay. HuCC-T1 cells were seeded at a density of 2 × 10^3 ^cells/well in 96-well plates with 100 μl of medium before addition of polymeric micelles. Next, free sorafenib, sorafenib-incorporated polymeric micelles, or empty polymeric micelles were added to 96-well plates at 100 μl. Controls were treated with 0.1% (*v*/*v*) of DMSO. Cells were incubated for 48 h, and cell viability was then measured in triplicate using an established MTT assay protocol.

## Results and discussion

### Characterization of sorafenib-incorporated Dex*b*LG nanoparticles

**Figure 1:**
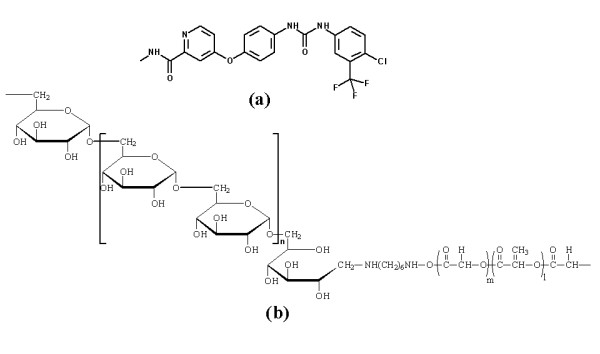
**Chemical structure of sorafenib (a) and Dex*b*LG copolymer (b)**.

Dextran is an appropriate macromolecule for block copolymerization because it has one reductive end. Dex*b*LG copolymer was synthesized by the coupling of aminated dextran and PLGA as described previously (Figure 1b) [13]. In the block copolymer, dextran acts as a hydrophilic domain and PLGA acts as a biodegradable hydrophobic domain. ^1^H NMR revealed the theoretical [MW_T_] and experimental [MW_E_] MWs of Dex*b*LG copolymer as 10,100 and 9,580 g/mol, respectively, while the MW of PLGA was 4,920 g/mol (*M*_w_) and 4,780 g/mol (*M*_n_) in gel permeation chromatography (data not shown). Because the Dex*b*LG copolymer is amphiphilic, it may form core-shell type nanoparticles in an aqueous environment [2,5,13]. In this configuration, dextran comprises the hydrophilic outer shell, while PLGA comprises the hydrophobic core that is the actual reservoir of sorafenib.

Sorafenib (Figure 1a) is a relatively novel class of angiogenesis inhibitor, which was selected as an anticancer drug due to its poor aqueous solubility. Nanoparticles are regarded as an ideal candidate for these kinds of drugs because the abundant microvascular structure of the tumor tissue is a useful target for nanoparticulate drug delivery system via the EPR effect.

**Figure 2:**
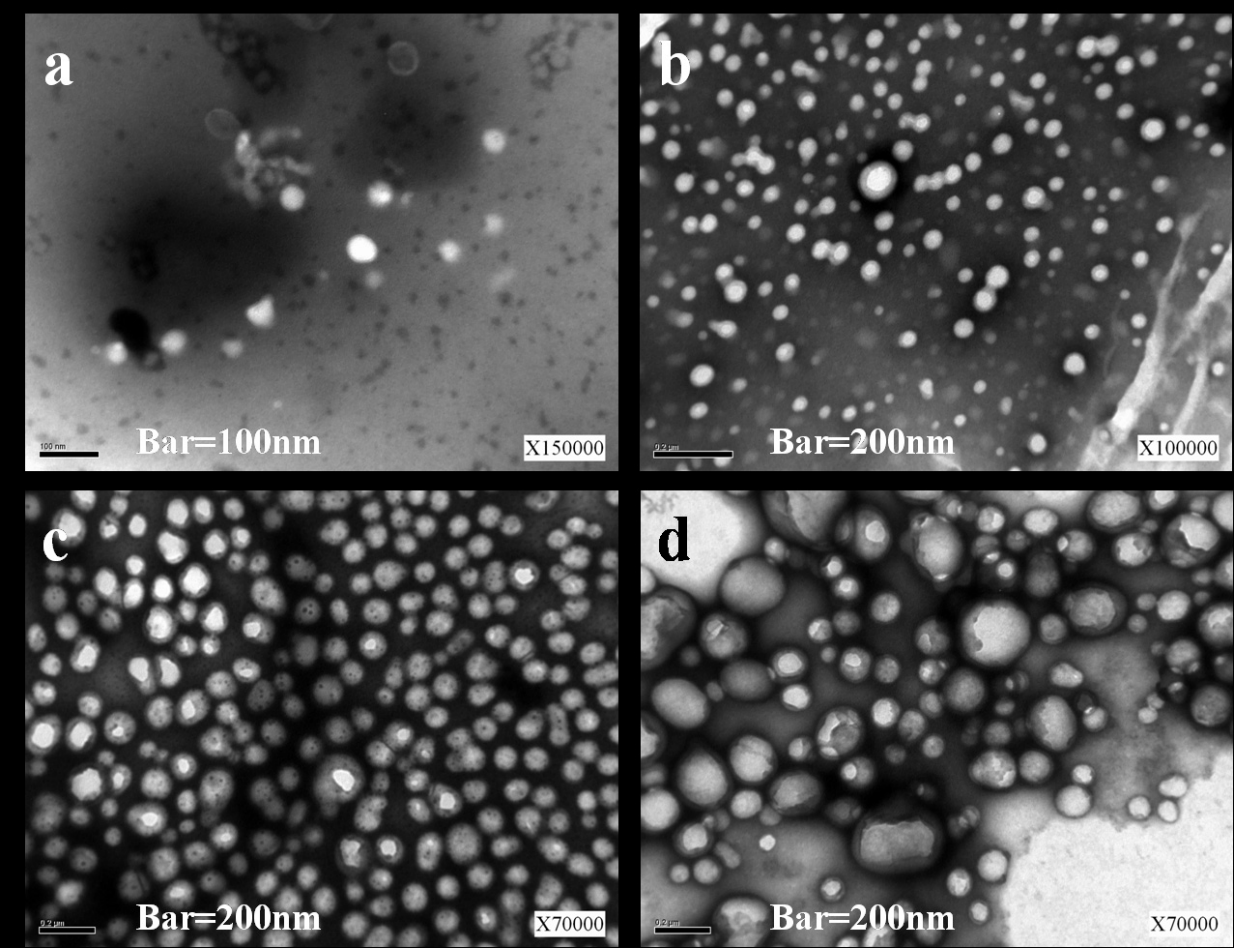
**TEM images of sorafenib-incorporated nanoparticles**. Polymer:drug empty nanoparticle (**a**), 40:2 (**b**), 40:5 (**c**), 40:7 (**d**) nanoparticles.

Nanoparticles of Dex*b*LG copolymer were prepared by nanoprecipitation-dialysis method [13]. Nanoprecipitation-dialysis method was superior over direct dialysis in minimizing particle size of nanoparticles (data not shown) [14]. Empty nanoparticles of Dex*b*LG had a spherical shape with a diameter < 100 nm (Figure 2a). As summarized in Table 1, their average particle size was 46 nm. Sorafenib-loaded Dex*b*LG nanoparticles were increased in size, with their size being related to the quantity of drug loaded (Figure 2b,d). Among them, nanoparticles with a polymer/dry weight ratio of 40:2 and 40:5 showed a relatively uniform size distribution in contrast to 40:7 nanoparticles. Particle size analysis of sorafenib-incorporated Dex*b*LG nanoparticles also showed an increased average particle size according to the contents of drug in the nanoparticles (Table 1). Zeta potential was not significantly changed according to drug incorporation. Drug contents were increased according to the feeding amount of drug (Table 1). Drug loading efficiency was lowest at 40:2, while 40:5 and 40:7 nanoparticles showed a loading efficiency > 35%.

**Table 1:** Characterization of sorafenib-incorporated nanoparticles

Polymer/drug (mg/mg)	Drug contents (%, *w*/*w*)		Loading efficiency (%, *w*/*w*)	Particle size (nm)	Zeta potential (mV)
	Theoretical	Experimental			
40:0	-	-	-	46 ± 1.12	-36.4 ± 3.1
40:2	4.76	1.23	25.84	63 ± 0.58	-35.5 ± 2.2
40:5	11.11	4.36	39.24	133 ± 0.58	-36.0 ± 1.1
40:7	14.89	5.26	35.33	181 ± 1.15	-35.8 ± 0.9

**Figure 3:**
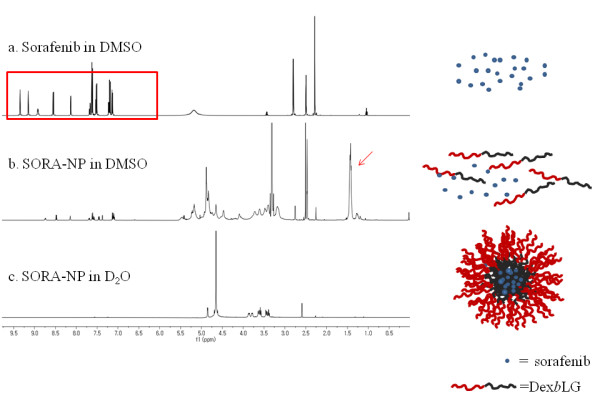
**1H NMR spectra**. Sorafenib in DMSO (**a**); SORA-NP, sorafenib-incorporated nanoparticles in DMSO (**b**); and SORA-NP in D_2_O (**c**). The box shows typical peaks of sorafenib (a), and the arrow shows typical peaks of PLGA (b).

To investigate the core-shell structure of nanoparticles and drug incorporation, ^1^H NMR was adapted to measure nanoparticles in DMSO-d_6 _or D_2_O (Figure 3). Sorafenib displayed intrinsic peaks in its^1^H NMR spectrum between 0.5 and 9.5 ppm (Figure 3a). When sorafenib-incorporated nanoparticles were reconstituted in D_2_O, only dextran peaks between 2.5 and 5.0 ppm were observed, while specific sorafenib and PLGA peaks at 1.45, 3.35, and 4.9 ppm disappeared (Figure 3c). However, all the peaks of sorafenib, dextran domain, and PLGA domain were apparent when nanoparticles were dissolved in DMSO (Figure 3b). These results indicated that sorafenib was successfully entrapped into the PLGA core of the nanoparticles and that the dextran domain constituted the hydrophilic outer shell.

**Figure 4:**
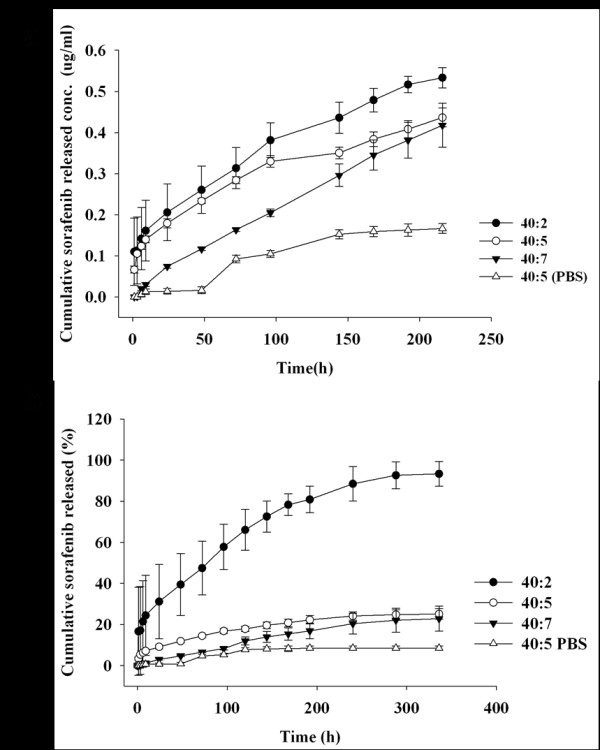
**Sorafenib release from the Dex*b*LG nanoparticles**. Time course of the absolute amount of released sorafenib (**a**) and total percentage of released sorafenib from Dex*b*LG nanoparticles (**b**). Drug release experiment of 40:2 (filled circle), 40:5 (empty circle), and 40:7 (inverted filled triangle) was performed with PBST, and 40:5 (PBS; empty triangle) was performed with PBS only.

Sorafenib release study was performed *in vitro*. Sorafenib release rate from 40:5 nanoparticles into PBST was significantly low, with < 10% of the total incorporated drug being released over 2 weeks (Figure 4). Therefore, PBST was used to maintain a sink condition in subsequent experiments. The sorafenib release rate changed according to the drug contents; increased drug incorporation produces slower release rates (Figure 4b). Notably, an initial burst of release from 40:2 nanoparticles was observed until 6 h, followed by a sustained release of sorafenib for 2 weeks. Unexpectedly, 40:5 and 40:7 nanoparticles showed a very low drug release for 2 weeks. This phenomenon might be due to the hydrophobic interaction at higher drug contents in the core of nanoparticles [2,13]. At higher drug contents, the hydrophobic drug might crystallize in the solid core of the nanoparticles, which would markedly hinder drug released [2]. However, when the absolute amount of released drug was compared, 40:2 and 40:5 nanoparticles showed an almost equal amount of drug release until 96 h (Figure 4a).

### *In vitro *cell cytotoxicity

**Figure 5:**
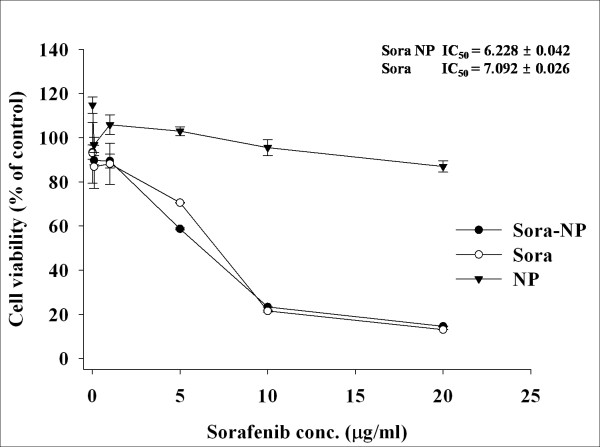
**Growth inhibition of HuCC-T1 cells by treatment of sorafenib-incorporated Dex*b*LG nanoparticles**. Two thousand cells were exposed to sorafenib, empty nanoparticles, and sorafenib-incorporated nanoparticles for 48 h.

Antitumor activity of sorafenib-incorporated Dex*b*LG nanoparticles was tested using HuCC-T1 cells. Sorafenib itself showed a dose-dependent antiproliferative effect against tumor cells that was almost the same as that produced by sorafenib-incorporated Dex*b*LG nanoparticles, while empty Dex*b*LG nanoparticles did not significantly inhibit cell viability (Figure 5). These results indicate that Dex*b*LG copolymer has no significant cytotoxicity against tumor cells, and sorafenib-incorporated Dex*b*LG has at least similar antitumor activity *in vitro*. Nanoparticles or nanomatrix was known to improve bioavailability [11] and antitumor activity *in vivo *[12] with a decreased hemolysis or myelosuppression effect. Our results demonstrate that Dex*b*LG nanoparticles are appropriate vehicles for sorafenib transport and release and that the nanoparticles are superior candidates for antitumor drug delivery.

## Conclusion

In this study, we prepared sorafenib-incorporated nanoparticles by nanoprecipitation-dialysis method. Sorafenib-incorporated Dex*b*LG nanoparticles adopt a spherical shape with a size < 300 nm. The higher the initial drug feeding, the higher is the quality of incorporated sorafenib. The size of the nanoparticles was increased according to the amount of sorafenib. Increasing quantity of incorporated sorafenib decreases the release rate of the drug. Sorafenib-incorporated Dex*b*LG nanoparticles have a similar antitumor activity against tumor cells *in vitro *compared to sorafenib itself. The collective results indicate the promise of sorafenib-incorporated Dex*b*LG nanoparticles as vehicles for antitumor drug targeting.

## Competing interests

The authors declare that they have no competing interests.

## Authors' contributions

DHK carried out the preparation of nanoparticles and drafted the manuscript. M-DK carried out the drug release studies. C-WC participated in the NMR analysis. C-WC participated in the analysis of drug contents and particle size. SHH participated in the observation of electron microscope. CHK participated in the cell viability assay. Y-HS designed the chemical structure of polymer. Y-IJ participated in the design of the study and coordination. DHK conceived the study and participated in its design and coordination. All authors read and approved the final manuscript.
